# Draft Genome Sequence of *Methanothermobacter* sp. Strain EMTCatA1, Reconstructed from the Metagenome of a Thermophilic Electromethanogenesis-Catalyzing Biocathode

**DOI:** 10.1128/genomeA.00892-17

**Published:** 2017-08-31

**Authors:** Hajime Kobayashi, Xiaohan Sun, Qian Fu, Haruo Maeda, Kozo Sato

**Affiliations:** a Department of Systems Innovation, School of Engineering, University of Tokyo, Tokyo, Japan; b Engineering for Sustainable Carbon Cycle (INPEX Corporation) Social Cooperation Program, Frontier Research Center for Energy and Resource (FRCER), School of Engineering, University of Tokyo, Tokyo, Japan; c INPEX Corporation, Tokyo, Japan

## Abstract

A draft genome of *Methanothermobacter* sp. strain EMTCatA1 was reconstructed from a metagenome of a thermophilic electromethanogenic biocathode. This genome will provide information about methanogens catalyzing methanogenesis at the biocathodes.

## GENOME ANNOUNCEMENT

Electromethanogenesis is a bioelectrochemical process at biologically catalyzed cathodes (biocathodes), in which CO_2_ is reduced into methane by using electrons from the electrodes (1). Hydrogenotrophic methanogens of the family *Methanobacteriaceae* have been found as the dominant archaea in most biocathode microbiotas and therefore are suggested to play a central role in catalyzing electromethanogenesis (2). To date, however, no genome of methanogen derived from the biocathode has been analyzed. Here, we report a draft genome of a methanogen, *Methanothermobacter* sp. strain EMTCatA1, which was reconstructed from shotgun sequences of a biocathode metagenome. The biocathode was inoculated with thermophilic microorganisms originating from deep subsurface water and could catalyze electromethanogenesis at a poised potential of up to −0.35 V versus the standard hydrogen electrode (SHE) (3).

DNA isolated from the biocathode was sequenced on the Illumina HiSeq 2000 platform (150-bp paired-end sequencing, two lanes), as described previously (4). Adapter and quality trimming of the reads was performed with Cutadapt version 1.8.3 (5). Approximately 395 million trimmed reads (ca. 60 Gb) were used for the metagenomics binning. The majority (~97%) of the sequences was assigned to two dominant species; 31% of the reads were assigned to an archaeal species (EMTCatA1), while 66% were assigned to a bacterial species (*Coriobacteriaceae* sp. strain EMTCatB1) (4). The reads were down-sampled to 400 Mb, thereby reducing sequences from relatively minor species, and assembled with Velvet (6). The contigs binned to strain EMTCatA1 were further assembled using Genetyx-Mac/ATSQ software (Genetyx, Tokyo, Japan), followed by gap filling with Sealer and quality checking with REAPR (7, 8). The resulting draft genome of strain EMTCatA1 is 1.72 Mb (G+C content, 49.6%) contained in a single circular scaffold with no gaps, representing a circular chromosome. The scaffold was annotated with Prokka (9), revealing a total of 1,856 features (1,814 protein-coding genes and 42 RNAs).

Phylogenetic analysis of the 16S and 23S rRNA genes and *mcrA* indicated that strain EMTCatA1 belongs to the genus *Methanothermobacter* of the family *Methanobacteriaceae*. The draft genome shares high similarities in sequence, gene content, and gene arrangement with the genomes of cultured members of the same genus (*Methanothermobacter thermautotrophicus* strain ΔH, *Methanothermobacter* sp. strain CaT2, and *Methanothermobacter marburgensis* strain Marburg) (10–12), suggesting that the ability to catalyze electromethanogenesis might be conserved among those methanogens. However, the cathode inoculated with a pure culture of *M. thermautotrophicus* strain ΔH showed no catalytic ability for the electrochemical reaction at potentials higher than −0.6 V versus the (3). Twenty genes in strain EMTCatA1 lack homologs in *M. thermautotrophicus* strain ΔH. It is possible that methanogens of the genus *Methanothermobacter* require one or more proteins encoded in those genes (including three putative membrane proteins and a ferredoxin-like protein), as well as certain conditions (e.g., acclimation to the cathode surface environment and the presence of other microorganisms) in order to effectively exhibit catalytic ability.

### Accession number(s).

The *Methanothermobacter* sp. strain EMTCatA1 draft genome reported here is available in the DDBJ/EMBL/GenBank databases under the accession number AP018336. The version described in this paper is the first version, AP018336.1.
